# Polymorphic microsatellite markers in the brown seaweed *Fucus vesiculosus*

**DOI:** 10.1186/s13104-015-1035-x

**Published:** 2015-03-08

**Authors:** Rui Candeias, Pilar Casado-Amezúa, Gareth A Pearson, Ester A Serrão, Sara Teixeira

**Affiliations:** Centre of Marine Sciences, CCMAR-CIMAR, University of the Algarve, Gambelas Campus, 8005-139 Faro, Portugal

**Keywords:** Intertidal, Genetic diversity, *Fucus vesiculosus*, Brown macroalga, Microsatellites

## Abstract

**Background:**

*Fucus vesiculosus* is a brown seaweed dominant on temperate rocky shores of the northern hemisphere and, is typically distributed in the mid-upper intertidal zone. It is an external fertilizer that reproduces sexually, providing an excellent model to address conflicting theories related to mating systems and sexual selection. Microsatellite markers have been reported for several *Fucus* species, however the genomic libraries from where these markers have been isolated, have originated from two or more species pooled together (*F. vesiculosus* and *F. serratus* in one library; *F. vesiculosus*, *F. serratus* and *Ascophyllum nodosum* in a second library), or when the genomic DNA originated from only one species it was from *Fucus spiralis*. Although these markers cross-amplify *F. vesiculosus* individuals, the level of polymorphism has been low for relatedness studies.

**Findings:**

The microsatellite markers described here were obtained from an enriched genomic library, followed by 454 pyrosequencing. A total of 9 microsatellite markers were tested across 44 individuals from the North of Portugal. The mean number of alleles across loci was 8.7 and the gene diversity 0.67.

**Conclusions:**

The high variability displayed by these microsatellite loci should be useful for paternity analysis, assessing variance of reproductive success and in estimations of genetic variation within and between populations.

## Findings

The seaweed genus *Fucus* (Fucaceae, Phaeophyta) dominates the intertidal biomass of the northern hemisphere’s shores, where several species co-occur and in some cases hybridize [1]. *Fucus vesiculosus* is a dominant producer on temperate rocky shores of the northern hemisphere, typically distributed in the mid-upper intertidal zone. It is dioecious, reproduces sexually, and has external fertilization, thus providing an excellent model to address conflicting theories related to mating systems and sexual selection. Variance in reproductive success can lead to sexual selection on reproductive traits and even to sexual conflict between the sexes [2]. Currently, nothing is known about the consequences of external fertilization and the role it plays in the evolution of reproductive traits in this species. Appropriate genetic markers, such as variable microsatellite loci, would allow for more detailed studies related to this topic as well as how mating systems influence patterns of variation within and between populations.

To date, four studies have reported microsatellite markers for different species of the *Fucus* genera [3-6]. However, none of the previously described markers have been isolated from the species *Fucus vesiculosus* alone, rather previous studies have been mainly focused on hybridization and speciation processes that affect several species of this genus. Therefore, genomic libraries with a mixture of two species or more have been developed to obtain common markers between the target species (*F. vesiculosus* and *F. serratus* in one library [6]; *F. vesiculosus*, *F. serratus* and *Ascophyllum nodosum* in a second library [3]). In the studies where the genomic DNA originated from only one species it has been from *Fucus spiralis* [4] and *Fucus guiryi* ([5], published as *F. spiralis* before this new species was described). Although these markers cross-amplify for *F. vesiculosus* individuals, their level of polymorphism has proven low for relatedness studies (unpublished observations, Teixeira S.).

Here we report the development and characterization of polymorphic microsatellite loci for *Fucus vesiculosus.* These markers will be useful for relatedness and population genetic studies of this species and hence to assess within and between population genetic diversity.

Whole genomic DNA was isolated from sperm cells of three *F. vesiculosus* males using the CTAB method [7]. We used sperm cells as starting material to avoid cross contamination from the abundant microbial communities commonly found in marine organisms. To isolate the microsatellite sequences, a combination of an SSR-enrichment protocol (standard CT/GT) with 454 pyrosequencing was performed by a commercial company (Ecogenics GmbH, Zürich, Switzerland). The same company designed 48 primers that we tested for polymorphisms across a panel of seven individuals obtained from locations in France (Roscoff [48°42′47.53″N; 4°02′32.95″W]) and Portugal (Viana do Castelo [41°41′32.57″N; 8°50′57.57″W]). An M13-tail (TGTAAAACGACGGCCAGT) was added at the 5′ end of all forward primers to enable fluorescent-dye labelling [8]. We identified nine polymorphic loci, and these results are presented in Table 1.

**Table 1:** **Characterization of the polymorphic microsatellite loci identified in** ***Fucus vesiculosus***

**Locus**	**Repeat motif**	**Primer sequence (5′-3′)**	**T**_a_ **(°C)**	**Allele size range (bp)**	***n***	***H***_E_	***H***_O_	***F***_IS_	**PIC**	***f*** **NL**
Fves1	(ATC)_9_	F: GCAGGATCGACAACCATACC	58	103-181	12	0.83	0.23	**0.7**	0.8	0.56
		R: GATCCCACCGACATGCTTTG								
Fves2	(AC)_12_	F: TGTATCAGCATACGGAAAAGC	56	100-112	7	0.76	0.63	0.16	0.71	0.06
		R: ATCAGGCATGTTGTCGTCGG								
Fves5	(TG)_11_	F: CGATGGGGGATGAGTGATTG	56	124-180	9	0.65	0.75	−0.15	0.6	0
		R: TGACATGCGGAGACAAAACG								
Fves7	(AC)_11_	F: ACTTGATGTCCAGAATGAATGGG	56	191-213	6	0.68	0.25	**0.6**	0.62	0.25
		R: AGGTGTGCGTGCGATTATTAC								
Fves8	(CA)_16_	F: TGGGGAAGTACACACACACG	56	137-169	6	0.50	0.4	**0.23**	0.45	0.09
		R: TCGTTGCGGTACTAACTTTGC								
Fves10	(TG)_11_	F: AGGCGCTTGAAAATGACCTG	56	103-169	6	0.47	0.2	**0.6**	0.44	0.4
		R: CCCGTGATTACTTTGGTATCGTC								
Fves11	(AC)_15_	F: TGTGTGACCTTCCTCCTGTG	56	133-147	5	0.45	0.43	0.04	0.41	0
		R: AGTATTGGCGTGTGCAACTG								
Fves12	(GT)_17_	F: AACTTTACCCGTTTTCCACG	58	107-159	18	0.82	0.85	−0.05	0.78	0
		R: CTCGCGGTAGAACACCTCTC								
Fves14	(AG)_17_	F: AATGACGGGGCCGGAATG	56	112-138	9	0.82	0.7	**0.19**	0.78	0.1
		R: GGCCGTCCGATCTTACTCAC								

The following details are reported: name, motif, primer sequence and annealing temperature (Ta°C). Also descriptive statistics are presented, based on one population analysed, number of alleles *n*, expected heterozygosity, *H*_E_, observed heterozygosity, *H*_O_, *F*_IS_ according to Hardy-Weinberg equilibrium, polymorphic information content, PIC, and null allele frequencies, *f* NL. Bold numbers indicate significant values at 5% level after q-value correction.

PCR amplification was performed in 10 μL reaction volumes containing 10 ng of genomic DNA, 1x Qiagen HotStart *Taq* buffer, 200 μM of dNTP’s, 0.04 μM of forward primer, 0.16 μM of reverse primer and fluorescently-labeled M13 primer, and 0.5 U of HotStart *Taq* polymerase (Qiagen). All amplifications were conducted in a Perkin-Elmer GeneAmp7200 (Waltham, MA, USA) with the following program: 15 min at 95°C; 30 cycles composed of 30 s denaturation at 95°C, 45 s at the annealing temperature (Table 1) and 45 s elongation at 72°C, followed by an additional 8 cycles composed of 30 s of denaturation at 95°C, 45 s at 53°C, 45 s elongation at 72°C, and a final extra elongation step for 30 min at 72°C. The forward primer for each set was fluorescently labelled with either FAM, ATT550, or HEX, and PCR products were multiplexed. Fragment analysis was conducted on an ABI 3130XL automated sequencer (Applied Biosystems, Foster City, CA, USA) with Rox350 size standard. Alleles were scored using Peak Scanner 1.0 (Applied Biosystems).

Genetic variation for all markers was tested across 44 individuals sampled in the North of Portugal (Viana do Castelo). The number of alleles per locus (*n*), expected (*H*_E_), observed heterozygosity (*H*_O_) and heterozygote deficiency (*F*_IS_; Table 1) were calculated using the software GENETIX 4.05 [9]. The polymorphic information content, PIC was calculated using PICcalc [10]. The majority of the optimized markers (9) were highly polymorphic, PIC values ranged from 0.41 (Fves11) to 0.8 (Fves1), the number of alleles found for the 9 loci ranged from 5 (Fves11) to 18 (Fves12) alleles; *H*_E_ varied from 0.45 (Fves11) to 0.83 (Fves1) and *H*_O_ from 0.2 (Fves10) to 0.85 (Fves12). Significant heterozygote deficiency was observed for 5 markers (Fves1, 7, 8, 10, and 14), as shown by the high and significant *F*_IS_ values (Table 1). Null alleles might occur at these loci, as confirmed by the determination of the frequency of null alleles in the dataset using ML- NULLFREQ [11] (Table 1). We tested for linkage disequilibrium between all pairs of loci using the software GENETIX 4.05 [9]. No linkage disequilibrium was found after the correction for multiple tests using the false discovery rate (FDR) approach [12] in QVALUE [13].

Previous studies found for the population of Viana do Castelo and across loci, gene diversities of 0.59 [5] and 0.58 [14], while the mean number of alleles found were 4.3 [5] and 5.6 [14]. The overall gene diversity found for the same population across loci, with our newly developed set of markers was of 0.67 and the mean number of alleles 8.7. The higher variability displayed by these microsatellite loci may be useful for paternity analysis and population genetic studies of this species.

### Availability of supporting data

The microsatellite sequences are available through the National Center for Biotechnology Information (see http://www.ncbi.nlm.nih.gov/). The accession numbers on the repository are the following: GenBank accession no. KP765803 through KP765811.
